# *Porphyridium purpureum* microalga physiological and ultrastructural changes under copper intoxication

**DOI:** 10.1016/j.toxrep.2021.04.015

**Published:** 2021-05-05

**Authors:** Zhanna V. Markina, Tatyana Yu. Orlova, Yuri A. Vasyanovich, Alexander I. Vardavas, Polychronis D. Stivaktakis, Constantine I. Vardavas, Manolis N. Kokkinakis, Ramin Rezaee, Eren Ozcagli, Kirill S. Golokhvast

**Affiliations:** a A.V. Zhirmunsky Institute of Marine Biology, National Scientific Center of Marine Biology, Vladivostok, 690041, Russia; b Far Eastern Federal University, Vladivostok, 690950 Russia; c Laboratory of Toxicology, School of Medicine, University of Crete, Heraklion, Greece; d Hellenic Mediterranean University, Department of Nutrition and Dietetics, Heraklion, Greece; e Clinical Research Unit, Faculty of Medicine, Mashhad University of Medical Sciences, Mashhad, Iran; f Neurogenic Inflammation Research Center, Mashhad University of Medical Sciences, Mashhad, Iran; g Istanbul University, Faculty of Pharmacy, Department of Pharmaceutical Toxicology, 34116, Beyazıt, Istanbul, Turkey; h Pacific Geografical Institite FEB RAS, Vladivostok, 690014, Russia; i N.I. Vavilov All-Russian Research Institute of Plant Genetic Resources, Saint Petersburg, Russia; j Siberian Federal Scientific Center of Agrobiotechnology RAS, Krasnoobsk, Russia

**Keywords:** Copper, *Porphyridium purpureum*, Rhodophyta, Fluorescence, Photosynthetic pigments, Reactive oxygen species, Ultrastructure, Aquatic pollution

## Abstract

The present work assessed the effect of copper (Cu) on cell dynamics and structure of the microalga *Porphyridium purpureum* (Rhodophyta, Bangiophycidae). Ultrastructure of the microalga was investigated and fluorescence of chlorophyll *a* and phycoerythrin, and content of reactive oxygen species (ROS) were estimated by flow cytometry. The number of cells did not show statistically significant differences at concentrations of 50 and 100 μg/L of Cu compared to the control, whereas 150 μg/L of Cu inhibited population growth. The fluorescence of chlorophyll *a* increased following exposure to Cu 100 μg/L and fluorescence of phycoerythrin enhanced by Cu 150 μg/L. There was no alteration in the above indicators at other concentrations. The content of ROS increased with increasing Cu concentration in a dose-dependent manner. The population size structure was also changed by Cu as the number of cells sized 4–6 μm was increased in the presence of Cu, especially with Cu 150 μg/L. Changes in the topography of thylakoids grew larger with Cu concentration.

## Highlights

• The number of cells did not differ significantly at Cu 50 and 100 μg/L compared to the control, whereas Cu 150 μg/L inhibited population growth.
• The fluorescence of chlorophyll a increased following exposure to Cu 100 μg/L and fluorescence of phycoerythrin enhanced by Cu 150 μg/L.
• The content of ROS increased with increasing Cu concentration in a dose-dependent manner.
• The population size structure was also changed by Cu as the number of cells sized 4−6 μm increased in the presence of Cu, especially with Cu 150 μg/L.

## Graphical abstract

**Figure:**
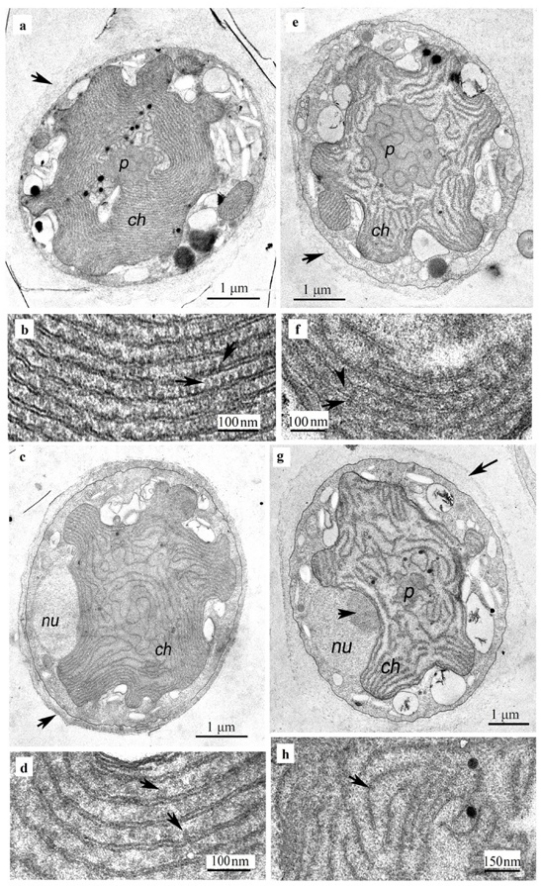

## 1 Introduction

Microalgae, as primary producers, have attracted the attention of researchers both in environmental and evolutionary terms. As the human impact on the environment increases, the number of studies devoted to assess the effects of toxic substances on such organisms, is growing [1]. Among the huge number of inorganic and organic substances, copper (Cu) plays a dual role, either assisting the growth of plants (acting as a cofactor of enzymes, working in the electron-transport chains of photosynthesis and respiration, ensuring the functioning of phytohormones, etc.) or at high concentrations, by inhibiting plants’ physiological and biochemical reactions it causes death [2,3]. Since Cu is commonly used in various industries, Cu contamination of aquatic systems is highly probable. Noteworthy, at high levels, Cu exerts cytotoxic properties and induces over-production of damaging free radicals. Microalgae have often been used to evaluate toxicity effects from various matrixes, such as carbon, carbon nanofibers, silicon nanotubes, oil-based biodiesels, fumes and nanoparticles, amongst others [[23], [24], [25], [26]].

The mechanisms underlying Cu effects on microalgae are fairly well described. However, information on the effect of Cu on representatives of marine algal flora and freshwater algae is limited to diatom species [4,5] and green microalgae, respectively [1,[6], [7], [8], [9], [10]]. Also, unicellular red algae remain almost unexplored [1,11]. Despite the fact that at the present stage of evolution, red algae have not been dominant in phytoplankton communities, studying their population and physiological responses to various toxic effects is very important to understanding the ecology of the phyla. Copper concentrations used in toxicity test can reach up to 1000 μg/L [1,4,7,11].

A quick method to qualitatively assess the state of microalgae cultures is via flow cytometry which is based on the entire arsenal of fluorescence-based methods of cell components and pathways/processes analysis [12].

Alterations in the number of microalgae cells are an indicator of the impact of environmental factors [9]. However, with a constant or increasing number of cells, their physiological processes can be inhibited.

The purpose of this work was to study changes in the number and size structure of the population, functioning of the photosynthetic apparatus, content of ROS, and ultrastructure of the microalgae species *Porphyridium purpureum* (Rhodophyta), in the presence of Cu. Thus, chlorophyll *a* fluorescence which reflects the functioning of the photosynthetic apparatus (the main energy supplier of plant cell) [3] was measured following exposure to Cu; also, reactive oxygen species (ROS) were quantified to evaluate Cu effects on a living organism, since one of the first responses to a stress factor is enhanced ROS production [2]. Besides, the microalgae ultrastructure was also examined following Cu treatment [7,9].

## 2 Material and methods

### 2.1 Microalgae culture

In this study, unicellular algae *P. purpureum* (Bory de Saint-Vincent) K.M. Drew & R. Ross (Rhodophyta), isolated from Peter the Great bay, a gulf near the southern coast of Primorsky Krai, East Russia, and the largest gulf of the Sea of Japan, were cultured. Until the 10th day of cultivation this strain does not form cell clusters.

The microalgae *P. purpureum* (Rhodophyta) strain MBRU_PP-AB11 were provided by the Marine Biobank resource center of the NSCMB FEB RAS (http://marbank.dvo.ru).

### 2.2 Toxicity test: general procedure

Algal culture in exponential growth phase was used for the inoculation of various Cu concentrations and control (the control group did not receive any additions of Cu). The experiments were carried out in three biological replicates, and the data are expressed as a percentage compared to the control group. The following endpoints were considered in this study: chlorophyll *a* and phycoerithrin fluorescence, and population growth (i.e. cell density).

The algae were grown on a medium *f* [13], prepared using filtered and sterilized seawater with a salinity of 32 % in 250 mL Erlenmeyer flasks with a culture medium volume of 100 mL [5], at a temperature of 18 °C, an illumination intensity of 70 μmol/m·s in the visible light region and a light/dark period of 14/10 h, respectively. The initial cell concentration was 10 × 10^4^ cells/mL. The duration of the experiment was 7 days. Samples needed for flow cytometric analysis, were taken after the 3rd and 7th day, whereas those required for electron microscopy analysis were collected after the 7th day.

### 2.3 Chemicals

Cu was added in the form of CuSO_4_ ‧5H_2_O, at concentrations (50, 100, and 150 μg/L) indicated in terms of Cu ions, and 2′,7′-dichlorofluorescin diacetate (Sigma-Aldrich) was used for ROS level measurement.

### 2.4 Flow cytometry

Microalgae cell counting and registration of morphological and biochemical changes during the experiment were carried out using the flow cytometer CytoFLEX (Beckman Coulter, USA) equipped with the software package CytExpert v.2.0. For cell number measurement, 10,000 events (particles in sample) were recorded at a flow rate of 60 μL/min. The selection of algae cells from the total number of events recorded by the cytometer, was carried out by the fluorescence of chlorophyll *a* [12]. The cell diameter was determined using calibration beads (Molecular Probes, USA) with the certified size distribution of 1, 2, 4, 6, 10 and 15 μm used for the forward scatter emission channel. The fluorescence intensity of chlorophyll *a* was detected at 690 nm (bandwidth 50 nm), and at 585 nm for phycoerythrin (bandwidth 20 nm), whereas the excitation wavelength was 488 nm for both pigments [12]. The level of ROS was evaluated using 2′7′-dichlorodihydrofluorescein diacetate fluorescent dye; staining was performed for one hour at room temperature in the dark. The fluorescence of the oxidized and diacetylated product was determined at 525 nm (bandwidth 20 nm) with 488 nm as the excitation wavelength [14]. After fluorescent staining, each sample was analyzed at a flow rate of 60 μL/min for 60 s.

### 2.5 Transmission electron microscopy

For electron microscopic analysis, *P. purpureum* cells were fixed for 2 h in 2.5 % glutaraldehyde prepared in a phosphate buffer (pH 7.4), and then in 1 % osmium tetroxide (in the same buffer) for 1 h. Afterwards, the material was dehydrated in a series of alcohols with increasing concentrations and acetone, and poured into a mixture of Epon and Araldite (Fluka, Switzerland) according to the standard procedure [15]. Sections with a thickness of 70 nm were made on an ultracutome Ultracut R LEICA (Austria) and contrasted with 2 % uranyl acetate and lead citrate solution according to the standard Reynolds method [16]. Sections were examined on a Libra 120 transmission electron microscope (TEM) (Carl Zeiss, Germany).

### 2.6 Statistical analysis

Statistical analyses of cell number and size, physiological parameters (chlorophyll *a* and phycoerythrin auto-fluorescence, fluorescence of 2′,7′-dichlorofluorescin diacetate product) were performed using Microsoft Excel with statistical significance set at a p-value <0.05. Differences in parameters among the different experimental conditions were assessed by non-parametric Mann–Whitney *U* test Results are expressed as mean ± standard error.

## 3 Results

The number of cells did not significantly differ from the control group in the presence of 50 and 100 μg/L of copper (Table 1, Table 2). However, addition of 150 μg/L of Cu to the medium, led to the inhibition of population growth.

**Table 1:** Growth and physiological parameters of *Porphyridium purpureum* in the presence of copper in the medium on the 3rd day.

Biomarkers	Copper concentrations			
	0 μg/L (control)	50 μg/L	100 μg/L	150 μg/L
Cell number in μL	269.06 ± 31.45	303.825 ± 50.06	262.04 ± 14.28	132.525 ± 2.93*
Chlorophyll *a* fluorescence, a.f.u.	95,926 ± 4170	95,400 ± 1805	110,449 ± 2416*	101,094 ± 5601
Phycoerythrin fluorescence, a.f.u.	60,016 ± 4170	60,219 ± 1805	62,783 ± 2416	69,216 ± 5602
Fluorescence of product of 2′,7′-dichlorofluorescin diacetate, a.f.u.	127,687 ± 3921	125,673 ± 1,017,288	108,862 ± 16,313*	126,957 ± 12,994

* Differences from the control group are significant at p ≤ 0.05.

**Table 2:** Growth and physiological parameters of the microalga *Porphyridium purpureum* in the presence of copper in the medium on the 7th day.

Biomarkers	Copper concentrations			
	0 μg/L (control)	50 μg/L	100 μg/L	150 μg/L
Cells number in μL	883.12 ± 35.84	843.55 ± 39.76	918.57 ± 90.06	571.21 ± 20.08*
Chlorophyll *a* fluorescence, a.f.u.	75,492 ± 5866	68,633 ± 4172	8,374,842 ± 3117*	7,414,917 ± 3711
Phycoerythrin fluorescence, a.f.u.	46,702 ± 4636	42,063 ± 3143	45,261 ± 2345	51,445 ± 3199
Fluorescence of product of 2′,7′-dichlorofluorescin diacetate, a.f.u.	39,818 ± 4636	39,551 ± 3143	45,296 ± 2345*	51,786 ± 3199*

* Differences from the control group are significant at p ≤ 0.05.

The fluorescence of chlorophyll *a* in Cu 50 and 150 μg/L treated culture did not significantly differ from the control group; but, that of culture treated with Cu 100 μg/L was higher than the control group (Table 1, Table 2). Phycoerythrin fluorescence following exposure to Cu 50 and 100 μg/L decreased compared to the control group at the end of the experiment but did show an increase at 150 μg/L of Cu (Table 1, Table 2).

The content of ROS in culture incubated with Cu 50 μg/L corresponded to that of the control group, while at 100 μg/L, it decreased on the 3rd day and exceeded the control group on the 7th. An even greater difference was noted in the presence of Cu 150 μg/L (Table 1, Table 2).

**Fig. 1:**
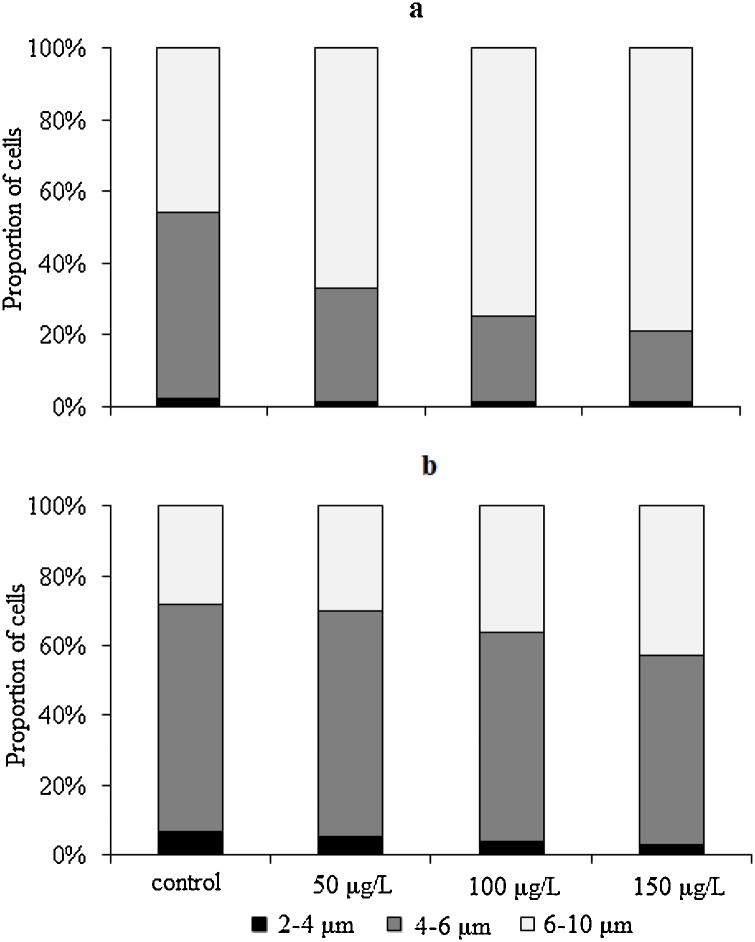
Size structure of the microalgae *Porphyridium purpureum* population in control and presence of copper in the medium. **A** – the third day; **B** – the seventh day.

The fraction of 6–10 μm cells on the 3rd day increased in the presence of various concentrations of Cu in the medium in a dose-dependent manner (Fig. 1a). On the 7th day, such an effect was observed only at concentrations of 100 and 150 μg/L (Fig. 1b).

**Fig. 2:**
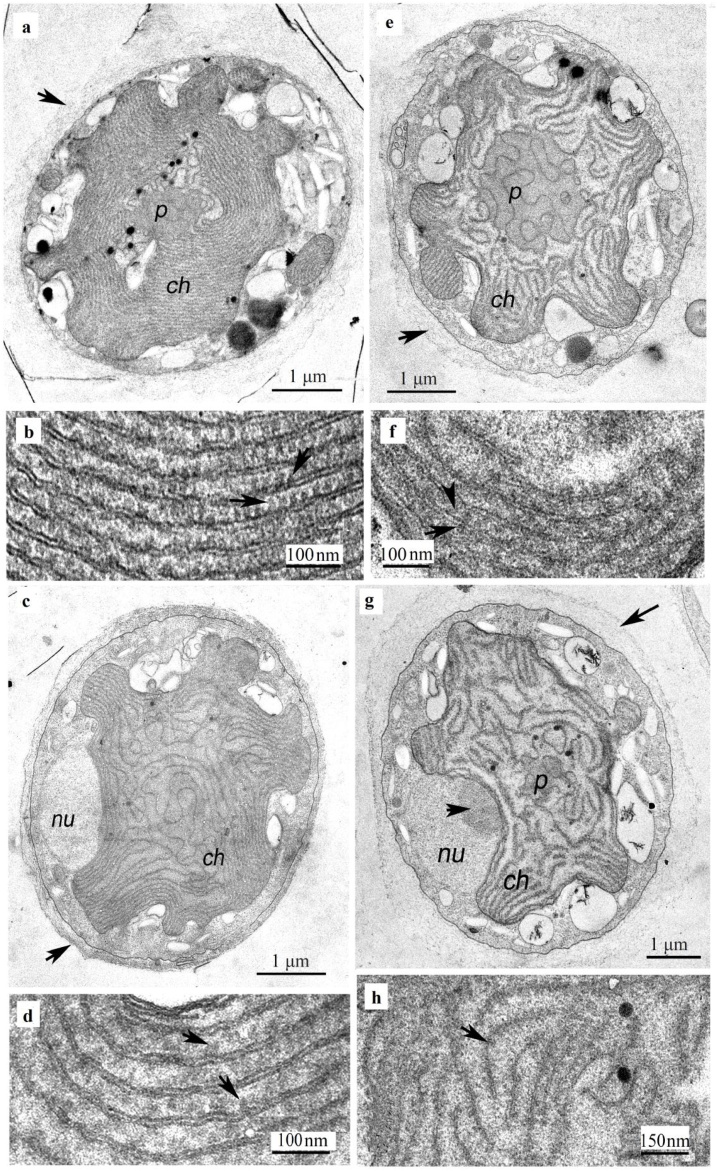
Ultrastructure of the microalga *Porphyridium purpureum* in presence of copper in the medium. **A** – control, general view, arrow indicates to mucopolysaccharide envelope; **B** – control, the location of thylakoids in the chloroplast: arrows indicate to phycobilisomes; **C** – copper content of 50 μg/L, general view, arrow indicates a mucopolysaccharide envelope; **D** – copper content of 50 μg/L, the location of thylakoids in the chloroplast: arrows indicate phycobilisomes; **E** – copper content of 100 μg/L, general view, arrow indicates a mucopolysaccharide mucous envelope; **F** – copper content of 100 μg/L, the location of thylakoids in the chloroplast: arrows indicate phycobilisomes; **G** – with a copper content of 150 μg/L, general view; nucleolus is indicated with a short arrow; the long arrow indicates the mucopolysaccharide mucous envelope; **H** – with a copper content of 150 μg/L, the location of thylakoids in the chloroplast; arrows indicate phycobilisomes. Designations: ***ch*** – chloroplast, ***p*** – pyrenoid, arrow indicates to mucopolysaccharide mucous envelope, ***nu*** – nucleus.

*P. purpureum* cells in the control group were spherical or ovoid, surrounded by a so-called “mucous envelope”, which forms due to the presence of pectin compounds. The nucleus with a diameter of 1–2 μm was oval in the peripheral part of the cell. Tubular mitochondria with a diameter of up to 0.5 μm, generally, occupied the peripheral part of the cell. A stellate chloroplast with a central pyrenoid was located in the central part of the cell (Fig. 2a). *P. purpureum* thylakoids were solitary, and located rather densely to each other. Phycobilisomes were arranged orderly and close to each other (Fig. 2b).

When exposed to Cu 50 μg/L, the ultrastructure of *P. purpureum* cells was similar to that of the control group. Chloroplasts underwent insignificant changes with thylakoids distanced from each other (Fig. 2c). Phycobilisomes, in general, were also arranged in an orderly manner, as in the control group (Fig. 2d).

Under the influence of Cu 100 μg/L, no violation of the cell ultrastructure was noted (Fig. 2e). However, the inter-thylakoid space in the chloroplasts increased even more than at a lower Cu concentration of 50 μg/L (Fig. 2f). Cu 150 μg/L did not alter the organelles of *P. purpureum* cells (Fig. 2g), with the exception of the distance of thylakoids in chloroplasts and the phycobilisomes that were arranged in a less orderly way (Fig. 2h).

## 4 Discussion

This study shows that the number of *P. purpureum* cells decreased following exposure to Cu 150 μg/L. In another red microalga species, *Rhodella reticulata*, this phenomenon occurred at 3 μg/L of Cu, along with a decrease in dry weight, and the content of chlorophyll *a*, carotenoids, and phycobilins [11]. The decrease in the number of cells discovered by the present study and previous studies, is due to the fact that Cu affects the growth of photosynthetic organisms by changing many cellular processes. Under stressful conditions, organisms dedicate the energy required for growth to other cellular processes necessary to control and maintain cell homeostasis and survival [6]. In addition, Cu has a negative effect on the morphology of chromosomes and the cell cycle, which leads to inhibition of cell division [17].

Cu upon entering the cytosol, disrupts the functioning of enzymes and cell division processes. The cell division of *P. purpureum* is susceptible to Cu than photosynthesis, with an increase in the proportion of larger cells. This is due to the fact that copper inhibits cell division processes regardless of carbon fixation (i.e. cells continue to produce substances necessary for building cells, but are not able to divide, which leads to an increase in their size due to the accumulated photosynthesis products) [4]. Cell enlargement is also considered one of the defence mechanisms that help microalgae adapt to induced toxic stress where under adverse conditions, algae can accumulate biomass, including even photosynthetic pigments. This process contributes to the “dilution” of toxicants in the metabolites secreted by the cell, hence, reducing the total adverse impact. In addition, as a result of an increase in cell size, their specific surface area also increases, which reduces the level of negative effects of substances [8].

One of the first protective measures employed by algae against toxic stress is production of mucous substances. This phenomenon has been widely shown.

Cells of *P. purpureum* possess a mucous envelope and do not expend energy for the realization of this detoxification mechanism. In addition, polysaccharides of red algae, bind to metal ions, causing the deposition of toxicants [11,18], and enhance the activity of antioxidant enzymes that suppress the effect of ROS [19].

The main damaging effect of Cu is due to the production of ROS due to their participation in the Fenton and Haber-Weiss reactions [3]. An increase in the ROS content in *P. purpureum* compared with the control group, was noted at Cu 100 μg/L and, to a greater extent, at 150 μg/L.

ROS are dangerous for the human body because they cause lipid peroxidation, violation of protein conformation and damage to nucleic acid molecules. It is known that phycoerythrin has antioxidant properties, like other phycobilines [20]; possibly, the synthesis of phycoerythrin increased following exposure to Cu 150 μg/L, as reflected by an increase in the level of pigment fluorescence.

Most of the active forms of oxygen are produced in chloroplasts and the mitochondria, and therefore these organelles are more susceptible to damage under toxic conditions [21]. As shown by ultrastructural assessments done in the present study, only the structure of chloroplasts underwent a change in the presence of Cu in the medium, whereas the mitochondria remained intact. At the same time, the fluorescence of photosynthetic pigments did not decrease, indicating adaptive rearrangements of the physiological processes of algae even with changes in the chloroplasts. On unicellular representatives of the Chlorophyta phyla [7,9] and red multicellular alga [18], a violation of the topography of thylakoids was also shown. Repeatedly on representatives of algae and higher plants like angiosperms, it was shown that chloroplasts are the target for different kinds of toxicants [2,22]. A change in the shape of chloroplasts can be a direct consequence of violation of ion exchange under the influence of metals, while changes in thylakoids are a consequence of the binding of their proteins to metals and changes in their normal functioning, as well as oxidative stress [3]. The main role of chloroplasts is their contribution to the photosynthesis process, but they also participate in the synthesis of amino acids and fatty acids and the immune responses of plants, therefore, damage to the photosynthetic apparatus inevitably affects the growth of algae [1].

Microalga *Phaeodactylum tricortum* was more sensitive to Cu then *P. purpureum* – after 3 days the cell number decreased 3-fold, but the cell number of *P. purpureum* did not differ significantly from the control over the same exposure time [4]. Also, *P. purpureum* was more tolerant to Cu than some species like *Isochrysis galbana* (50 % growth inhibition after 3 days were observed under 10–18 μg/L) and *Phaeocystis antarctica* (50 % growth inhibition after 10 days were observed under 6 μg/L) [1].

## 5 Conclusions

Cu 50 μg/L and 100 μg/L did not affect the dynamics of *P. purpureum* cell numbers; however, Cu 150 μg/L inhibited the growth of the algae. Chlorophyll *a* and phycoerythrin fluorescence in Cu 100 μg/L and 150 μg/L exposed cells, exceeded that of the control group, respectively. At the end of the treatment period, ROS content increased following exposure to Cu 100 μg/L and 150 μg/L. The size structure of the population changed during copper intoxication and ultrastructural changes were only observed for chloroplasts. Adaptation of *P. purpureum* to Cu 50 μg/L in the medium occurred by the seventh day, as indicated by the correspondence of all parameters to the control level, however, the chloroplast ultrastructure was not restored. At 100 μg/L, the number of cells did not differ from that of the control group, however, the size structure of the population changed, and there was an increase in the content of ROS as well as even greater violations in the chloroplasts. Cu 150 μg/L led to inhibition of growth processes, an even greater increase in the content of ROS and more marked changes in the topography of thylakoids.

## Authors statement

Zhanna V. Markina: Methodology and data collection and analysis

Tatyana Yu. Orlova: Methodology and data collection and analysis

Yuri A. Vasyanovich: Methodology and data collection and analysis

Alexander I. Vardavas: Data analysis and manuscript drafting

Polychronis D. Stivaktakis: Data analysis and manuscript drafting

Constantine I. Vardavas: Data analysis and manuscript drafting

Manolis N. Kokkinakis, Ramin Rezaee, Eren Ozcagli, Critical Reviewing and Editing,

Kirill S. Golokhvast: Conceptualization and Supervision

## Conflict of Interest

The authors declare no conflict of interest.

## References

[1] Miazek K., Iwanek W., Remacle C., Richel A., Goffin D. (2015). Effect of metals, metalloids and metallic nanoparticles on microalgae growth and industrial products biosynthesis: a rewiew. Int. J. Mol. Sci..

[2] Yruela I. (2005). Copper in plants. Braz. J. Plant Physiol..

[3] Nagajyoti P.S., Li K.D., Sreekanth T.V.M. (2010). Heavy metals, occurrence and toxicity for plants: a review. Environ. Chem. Lett..

[4] Cid A., Fidalgo P., Herrero C., Abalde J. (1996). Toxic action of copper on the membrane system of a marine diatom measured by flow cytometry. Cytom. Part A.

[5] Araújo C.V.M., Diz F.R., Lubián L.M., Blasco J., Moreno-Garrido I. (2010). Sensitivity of *Cylindrotheca closterium* to copper: influence of three test endpoints and two test methods. Sci. Total Environ..

[6] Perales-Vela H.V., González-Moreno S., Montes-Horcasitas C., Cañizares-Villanueva R.O. (2007). Growth, photosynthetic and respiratory responses to sub-lethal copper concentrations in *Scenedesmus incrassatulus* (Chlorophyceae). Chemosphere.

[7] Chen Z., Song S., Wen Y., Zou Y., Liu H. (2016). Toxicity of Cu (II) to the green alga *Chlorella vulgaris*: a perspective of photosynthesis and oxidant stress. Environ. Sci. Pollut. Res. Int..

[8] Machado M.D., Soares E.V. (2014). Modification of cell volume and proliferative capacity of *Pseudokirchneriella subcapitata* cells exposed to metal stress. Aquat. Toxicol..

[9] El-Naggar A.H., Sheikh H.M. (2014). Response of the green microalga *Chlorella vulgaris* to the oxidative stress caused by some heavy metals. Life Sci. J..

[10] Yang J., Cao J., Xing G., Yuan H. (2015). Lipid production combined with biosorption and bioaccumulation of cadmium, copper, manganese and zinc by oleaginous microalgae *Chlorella minutissima* UTEX2341. Bioresour. Biotechnol..

[11] Toncheva-Panova T., Merakchiyska M., Djingova R., Ivanova J., Sholeva M., Paunova S. (2006). Effect of Cu^2+^ on the red microalga *Rhodella reticulata*. Gen. Appl. Plant Physiol..

[12] Hyka P., Lickova S., Přibyl P., Melzoch K., Kovar K. (2013). Flow cytometry for development of biotechnological processes with microalgae. Biotechnol. Adv..

[13] Guillard R.R.L., Ryther J.H. (1962). Studies of marine planktonic diatoms. 1. *Cyclotella nana* Hustedt, and *Detonula confervacea* (Cleve) Gran. Can. J. Microbiol..

[14] Gomes F., Fernandes E., Lima J.L. (2005). Fluorescence probes used for detection of reactive oxygen species. J. Biophys. Biochem. Methods..

[15] Luft J.H.J. (1961). Improvements in epoxy resin embedding methods. J. Biophys. Biochem. Cytol..

[16] Reynolds E. (1963). The use of lead citrate at high pH as an electron-opaque stain in electron microscopy. J. Cell Biol..

[17] Jiang W., Liu D.H., Liu X. (2001). Effect of copper on root growth, cell division, and nucleolus of *Zea maye*. Biol. Plant..

[18] Gouveia C., Kreusch M., Schmidt E.S., de L. Felix M.R.L., Osorio L.K.P., Pereira D.T., Santos R., Ouriques L.C., Martins R.P., Latini A., Ramlov F., Carvalho T.J.G., Chow F., Maraschin M., Bouzon Z.L. (2013). The effects of lead and copper on the cellular architecture and metabolism of the red alga *Gracilaria domingensis*. Microsc. Microanal..

[19] Sun L., Wang C., Shi Q., Ma C. (2009). Preparation of different molecular weight polysaccharides from *Porphyridium cruentum* and their antioxidant activities. Int. J. Biol. Macromol..

[20] Hemlata V., Sumbul A., Tasneem F. (2018). Extraction, purification and characterization of phycoerythrin from Michrochaete and its biological activities. Biocatal. Agric. Technol..

[21] Schmitt F.-J., Renger G., Friedrich T., Kreslavski V.D., Zharmukhamedov S.K., Los D.A., Kuznetsov V.V., Allakhverdiev S.I. (2014). Reactive oxygen species: re-evaluation of generation, monitoring and role in stress-signaling in phototrophic organisms. Biochim. Biophys. Acta Bioenerget..

[22] Andosch A., Affenzeller M.J., Lütz C., Lütz-Meindl U. (2012). A freshwater green alga under cadmium stress: ameliorating calcium effects on ultrastructure and photosynthesis in the unicellular model *Micrasterias*. J. Plant Physiol..

[23] Pikula K.S., Zakharenko A.M., Chaika V.V., Vedyagin A.A., Orlova T.Y., Mishakov I.V., Kuznetsov V.L., Park S., Renieri E.A., Kahru A., Tsatsakis A.M., Golokhvast K.S. (2018). Effects of carbonand silicon nanotubes and carbon nanofiberson marine microalgae Heterosigma akashiwo. Environ. Res..

[24] Pikula K.S., Stratidakis A., Zakharenko A.M., Sarigiannis D., Rakitskii V.N., Hayes W.A., Coleman M.D., Waissi G., Kokkinakis M., Chaika V.V., Tsatsakis A.M., Golokhvast K.S. (2019). Toxicity bioassay of waste cooking oil-based biodiesel to marine microalgae. Toxicol. Rep..

[25] Pikula K., Kirichenko K., Zakharenko A., Chaika V., Markina Zh., Orlova T., Waissi G., Kholodov A., Tsatsakis A., Golokhvast K. (2019). Dependence of welding fume particle toxicity on electrode type and current intensity assessed by microalgae growth inhibition test. Environ. Res..

[26] Pikula K., Mintcheva N., Kulinich S.A., Zakharenko A., Markina Z., Chaika V., Orlova T., Mezhuev Y., Kokkinakis E., Tsatsakis A., Golokhvast K. (2020). Aquatic toxicity and mode of action of CdS and ZnS nanoparticles in four microalgae species. Environ. Res..

